# Emotional State of Chinese Healthcare Workers During COVID-19 Pandemic

**DOI:** 10.3389/fpsyg.2022.854815

**Published:** 2022-03-23

**Authors:** Minggang Jiang, Xu Shao, Shengyi Rao, Yu Ling, Zhilian Pi, Yongqiang Shao, Shuaixiang Zhao, Li Yang, Huiming Wang, Wei Chen, Jinsong Tang

**Affiliations:** ^1^Department of Psychiatry, The Fourth People’s Hospital of Jiande, Hangzhou, China; ^2^Department of Psychiatry, Sir Run Run Shaw Hospital, Zhejiang University School of Medicine, Hangzhou, China; ^3^Department of Public Health, Health Bureau of Jiande City, Hangzhou, China

**Keywords:** mental health, healthcare workers, depression, COVID-19, anxiety

## Abstract

**Objective:**

Anti-epidemic work against coronavirus disease (COVID) has become routine work in China. Our study was intended to investigate the emotional and psychological state of healthcare workers and look for the association between sociodemographic factors/profession-related condition and emotional state.

**Methods:**

A cross-sectional survey was conducted online among healthcare workers from various backgrounds. Symptoms of anxiety and depression were assessed by the Chinese versions of the seven-item Generalized Anxiety Disorder (GAD-7) and the nine-item Patient Health Questionnaire (PHQ-9), respectively. Supplementary questions (Supplementary Material) were recorded to describe the participants’ information about workplace violence, profession, and attitude related to the COVID pandemic. Wherever suitable, independent *t*-test, and one-way ANOVA were performed to detect group differences of GAD-7 and PHQ-9 total scores after grouping by sociodemographic variables, respectively, such as age, gender, marital status, educational level, after-tax income, department category, job title, experience of workplace violence, and anti-epidemic participation. Multiple linear regression analyses (stepwise method) were utilized in order to look for the potential associated factors of GAD-7 and PHQ-9 total scores.

**Results:**

A total of 2,139 questionnaires with valid response were completed. Approximately 86.44% of participants had minimal symptoms of anxiety, 11.08% mild, 1.59% moderate, and 0.89% severe. Meanwhile, 81.34% had minimal symptoms of depression, 14.07% mild, 2.90% moderate, 1.17% moderately severe, and 0.51% severe. Student’s *t*-test showed that participants with female gender, with experience of workplace violence scored higher on both GAD-7 and PHQ-9, and participants with experience of anti-epidemic front-line work during pandemic scored lower on both GAD-7 and PHQ-9. ANOVA showed that participants aging from 31 to 40, with higher educational level, with middle level of annual after-tax income, with department of internal medicine or surgery, or with middle level of job title scored higher on both GAD-7 and PHQ-9. Regression analyses showed that female gender, high job title, and the experience of workplace violence positively were associated with anxiety or depression. Doctoral education, department (other *vs*. psychiatry), job enthusiasm, and professional self-identity were negatively associated with anxiety or depression. Additionally, psychological support was negatively associated with depression.

**Conclusion:**

As the epidemic prevention and control against COVID-19 become normalized in China, emotional state of healthcare workers deserves extensive attention. Our study revealed that gender, educational level, department category, job title, the experience of workplace violence, job enthusiasm, and professional self-identity are the most important influencing factors of physician’s anxiety and depression. Self-tailored psychological intervention should be based on the predisposing factors above to mentally prepare healthcare workers for this long-lasting battle against COVID-19.

## Introduction

Since the beginning of 2020, the outbreak of the coronavirus disease 2019 (COVID-19) pandemic has become a public health emergency that caused international concern (WHO, 2020). The COVID-19 has a high incidence, strong infectivity, and certain mortality, which seriously threatened the life and health of all mankind (Hui et al., 2020). As this epidemic is not likely to end any time soon, healthcare workers worldwide are undergoing a long-last battle against COVID-19. During the pandemic, the United Nations already highlighted that front-line healthcare workers had a considerable vulnerability to having mental health needs (United Nations, 2020). Compared to other populations, healthcare workers are at great risk of exposure to COVID-19, thus faced with a tremendous level of stress (Chen et al., 2020; Shanafelt et al., 2020). Also, healthcare workers have to witness patients dying alone and then notify this traumatic affair to families, which could result in excessive stress and burnout (Yin et al., 2020). Under this heavy psychological stress, a study reported that 28.6% of healthcare workers suffered from moderate to severe mental disturbances, with young women affected the most (Kang et al., 2020). The psychological burden and overall wellness of healthcare workers have received huge awareness, with research showing high rates of burnout, psychological stress, and suicide (Santarone et al., 2020). Therefore, the mental health status of healthcare workers is worthy of investigation during this global pandemic.

For fear of infection or death during the outbreak, healthcare workers may experience various acute psychological effects, such as symptoms of anxiety and depression (Bao et al., 2020). Epidemiological research in China has reported that approximately 11–50% of healthcare workers reported significant anxiety symptoms (Lai et al., 2020; Liu C. Y. et al., 2020; Liu Q. et al., 2020), and approximately 43–50% of Chinese healthcare workers reported significant depressive symptoms (Lai et al., 2020; Zhu et al., 2020).

And several studies have been performed to look for the predisposing factors of anxiety and depression among healthcare workers. Gender studies found that compared to male, women during the COVID-19 pandemic were more likely to experience anxiety (Islam et al., 2020, 2021) and depression (Sudha et al., 2018; Banna et al., 2020; González-Sanguino et al., 2020; Islam et al., 2020, 2021). Healthcare workers aging from 31 to 40 had higher anxiety and depression than other age groups (Jagiasi et al., 2021). Being married was risk factor of anxiety, not depression (Liu et al., 2021). Healthcare workers in departments responsible for care of COVID-19 patients (i.e., department of emergency, intensive care unit, infections disease) had greater likelihood of developing anxiety and depression than other departments (Lai et al., 2020; Lu et al., 2020). Intermediate technical title was associated with severe anxiety and depression (Lai et al., 2020). Additional analyses revealed that healthcare workers with educational degree lower than doctor had significantly higher anxiety and depression than those with doctoral degree (Elliott et al., 2021). These results have suggested that the development of emotional issues among healthcare workers during this pandemic is related to multiple sociodemographic factors, such as gender, age, marital status, department, job title, and educational level.

The emotional state may also be influenced by many psychosocial factors characteristic of the Chinese medical environment. First, the workplace violence against healthcare workers has been a serious public problem in China (Ma et al., 2021), and how this kind of act changes during the pandemic is meaningful to find out. Second, Chinese primary healthcare workers are not satisfied with job welfare and income (Sang et al., 2022), often excessive devotion with mismatching reward. No study so far has investigated the association between workplace violence/salary satisfaction and emotional issues. Third, during the pandemic, Chinese public media has made wide propaganda for healthcare workers selflessly shouldering the responsibility of saving lives, but how this social media exposure influences healthcare workers’ emotional states remains unknown. Previous studies showed that a lack of social or emotional support was associated with anxiety or depression (Jagiasi et al., 2021) and that social support for medical staff was negatively associated with anxiety and stress (Xiao et al., 2020). Considering this, media publicity may be a protective factor of emotional issues.

Besides the psychosocial factors above, vocational evaluation is also a crucial factor worthy of investigation. Based on the experience of previous epidemics, healthcare workers would expect recognition from the health authorities (Koh et al., 2005; Khalid et al., 2016). According to a study during the outbreak in China, the most important factors which motivate the healthcare workers to continue working were social and moral responsibilities and professional obligation (Cai et al., 2020). Along with uncertainty and burden in the workplace, healthcare workers are at the risk for reduced perception of work accomplishment, negative attitude toward work, and disengagement from work (Demerouti et al., 2003; Malach-Pines, 2005; Albott et al., 2020). Healthcare workers may also suffer from stigmatization (Schubert et al., 2021), consequently reducing self-esteem and self-efficacy (Corrigan et al., 2006). These studies have shown a dramatic shift of vocational evaluation during this pandemic, such as job satisfaction and career identity, but how it contributes to healthcare workers’ emotional states remains unknown, thus calling for further investigation.

This study was designed to fully capture the effect of different sociodemographic variables and vocational evaluation on emotional states of anxiety or depression under the environment of COVID-19 pandemic. We delivered an online survey among healthcare workers in Jiande City, Zhejiang Province, China, and recorded sociodemographic data and screening scores of the seven-item Generalized Anxiety Disorder Questionnaire (GAD-7) and the nine-item Patient Health Questionnaire (PHQ-9). Supplementary questions (Supplementary Material) were asked mainly about vocational evaluation, and other work-relation conditions were also recorded such as workplace violence, anti-epidemic participation, and psychological resource. We hypothesize that: (1) participants with female gender, from the department with a high risk of medical exposure or with a low educational level score higher on GAD-7 and PHQ-9; (2) negative vocational evaluation was associated with high scores of GAD-7 and PHQ-9.

## Materials and Methods

### Participants

Healthcare workers from public hospitals in Jiande City, Zhejiang Province, China, took part in the current study. There are altogether 21 public hospitals in the whole city, such as municipal hospitals, community healthcare centers, and township health centers, with over 3,000 healthcare workers. Altogether, 2,190 participants answered the whole survey, and the response rate was over 70%. Data of 51 individuals were ruled out due to invalid responses. Among the remaining 2,139 participants, there were 584 men (27.30%) and 1,555 women (72.70%). The mean age of this population was 34.99 years ± 9.34 *SD*, with age ranging from 20 to 60 years. This study was conducted in accordance with the Declaration of Helsinki. The study protocol was approved by a local ethics committee (Ethics Committee of the Fourth People’s Hospital of Jiande City, No. 2020002-05) and all participants gave their digital informed consent.

### Questionnaire Measures and Procedure

This survey was designed to investigate the emotional and psychological states of healthcare workers during this COVID-19 pandemic. Questionnaires were delivered online from December 2020 to January 2021, and participants filled out the survey *via* electronic devices (e.g., mobile phone, laptop, etc.). The questionnaire link was disseminated *via* WeChat, the most commonly used instant messenger in China. Sociodemographic data were collected, namely age, gender, marital status, educational level, after-tax income, department category, job title, and working age. The screening tools of emotional issues and psychosocial questions were described as follows.

#### The Seven-Item Generalized Anxiety Disorder Questionnaire

The GAD-7 is a brief self-report measure detecting generalized anxiety disorder (GAD) based on the Diagnostic and Statistical Manual of Mental Disorders, Fourth Edition (DSM-IV) (American Psychiatric Association, 2000) symptom criteria. Each of the seven items asks individuals how often each symptom bothered them during the past 2 weeks. The total score ranges from 0 to 27, with response option on each item ranging from “not at all” (0 point) to “nearly every day” (three point). A score of 10 or greater on the GAD-7 represents a reasonable cut point for identifying cases of GAD. Cut points of 5, 10, and 15 are interpreted, respectively, as representing mild, moderate, and severe levels of anxiety on the GAD-7. This instrument was proved to have good internal and test-retest reliability, as well as convergent, construct, criterion, procedural, and factorial validity (Spitzer et al., 2006). The Chinese version of the GAD-7 was first introduced and validated among general hospital outpatients in 2010, and an optimal cutoff point of 10 was replicated (He et al., 2010). The internal reliability was 0.93 in the current study.

#### The Nine-Item Patient Health Questionnaire

The PHQ-9 is the nine-item depression module from the full Patient Health Questionnaire (PHQ) (Spitzer et al., 1999). The PHQ-9 is a structure-validated self-report questionnaire commonly utilized for identifying potential people with depression based on the DSM-IV symptom criteria for a major depressive episode (Kroenke et al., 2001). As a severity measure, the total score ranges from 0 to 27, with response option on each item ranging from “not at all” (zero point) to “nearly every day” (three point). A score of 10 or greater on the PHQ-9 represents a reasonable cut point for identifying cases of major depression. Cut points of 5, 10, 15, and 20 are interpreted, respectively, as representing mild, moderate, moderately severe, and severe levels of depression. The PHQ-9 has been validated across various Chinese populations showing stably satisfactory feasibility, reliability, and validity (Chen et al., 2010, 2013; Liu et al., 2011; Yu et al., 2012; Zhang et al., 2013). The internal reliability was 0.93 in the current study.

#### Psychosocial Questions Related to Vocation During COVID-19 Pandemic

As are listed in Supplementary Material, the first variable was named as the experience of workplace violence, with seven yes-or-no items asking individuals the experiences of workplace violence during the past 12 months. The total score ranges from 0 to 7, with a higher score indicating more experiences of violence. The second variable was named as salary satisfaction, with one single-choice item asking individuals about their salary satisfaction. The third variable was named as anti-epidemic participation, with one yes-or-no item asking individuals whether they have participated in front-line work against COVID-19. The fourth variable was named as media publicity, with one five-point item asking how individuals feel about publicity for healthcare workers during the pandemic. The fifth variable was named as job enthusiasm, with eleven items with five-Likert point measuring individuals’ job enthusiasm and motivation during the pandemic. The total score ranges from 11 to 55, with a higher score indicating greater willingness to devote oneself in medical and anti-epidemic activities. The sixth variable was named as professional self-identity, with thirteen items with seven-Likert point measuring to what extent healthcare workers embrace and accept their profession and lives built upon it. Total score ranges from 13 to 91, with a higher score indicating a greater sense of professional self-identity. The last variable was named psychological support, with one multiple-choice item counting how many kinds of mental health service healthcare workers had received from the work unit during the pandemic.

### Statistical Methods

Continuous variables were summarized as mean ± SD, and categorical variables as number (percentage). Independent *t*-test was performed to detect group differences of GAD-7 and PHQ-9 total scores after grouping the study sample based on gender, anti-epidemic participation, along with violence experience (transformed into binary variable), respectively. One-way ANOVA was performed to detect group differences of GAD-7 and PHQ-9 total scores after grouping the study sample based on age (transformed into categorical variable), marital status, educational level, after-tax income, department category, and job title, respectively. Whenever a significant main effect was found, *post-hoc* multiple testing correction was conducted using Bonferroni adjustment to evaluate between-group differences. More importantly, multiple linear regression analyses (stepwise method) were utilized in order to look for the potential associated factors of GAD-7 and PHQ-9 total scores, with sociodemographic variables and variables derived by Supplementary questions (Supplementary Material) as independent factors. In regression, dummy variables were set for unordered polytomous variables such as marital status (*vs*. unmarried), educational level (*vs*. junior college), and department category (*vs*. psychiatry). All statistical analyses were carried out using SPSS, version 26 (SPSS Inc., Chicago, IL, USA). A *p*-value < 0.05 was considered significant for statistical tests.

## Results

### Sociodemographic Features and Survey Scores of the Whole Sample

Among the 2,139 subjects who filled in the questionnaire, the distribution was not uniform. In our study sample, 70.59% aged from 31 to 40 years, 72.70% were female, 38.34% were unmarried, 67.84% earned bachelor degree, 65.73% had annual after-tax income of 60,000 to 120,000 yuan, 45.63% were from other departments (e.g., ultrasound, radiology, rehabilitation, laboratory, and pharmacy), and 55.68% earned primary job title. On average, participants had over 13 years of working age. See Table 1 for details.

**TABLE 1:** Sociodemographic features of the study sample (*N* = 2,139).

Demographic variables	*N* (%)
**Age (years)**	
20–30	554 (25.90%)
31–40	1,510 (70.59%)
41–50	69 (3.23%)
51–60	6 (0.28%)
**Gender**	
Male	584 (27.30%)
Female	1,555 (72.70%)
**Marital status**	
Unmarried	820 (38.34%)
Married	708 (33.10%)
Divorced	445 (20.80%)
Widowed	166 (7.76%)
**Educational level**	
Junior college	511 (23.89%)
Bachelor	1451 (67.84%)
Master	87 (4.07%)
Doctor	2 (0.93%)
Others	88 (4.11%)
**Annual after-tax income (Chinese yuan)**	
≤60,000	336 (15.71%)
60,000–120,000	1406 (65.73%)
120,000–200,000	381 (17.81%)
≥200,000	16 (0.75%)
**Department category**	
Internal medicine	615 (28.75%)
Surgery	327 (15.29%)
Gynecology and Pediatrics	164 (7.67%)
Psychiatry	57 (2.66%)
Others	976 (45.63%)
**Job title**	
Primary title	1191 (55.68%)
Middle title	649 (30.34%)
Vice senior title	223 (10.43%)
Senior title	76 (3.55%)
Working age (years, M ± S.D.)	13.67 ± 9.52

*N, number of participants; M, mean value; S.D., standard deviation.*

Among all participants, mean score of GAD-7 was 1.57 ± 2.95, with 2.48% of participants screened positive (total score ≥ 10) for generalized anxiety disorder. Approximately 86.44% had minimal anxiety, 11.08% mild, 1.59% moderate, and 0.89% severe. Mean score of PHQ-9 was 2.19 ± 3.80, with 4.58% of participants screened positive (total score ≥ 10) for depressive disorder. 81.34% had minimal depression, 14.07% mild, 2.90% moderate, 1.17% moderately severe, and 0.51% severe. In terms of supplementary questions, 75.13% experienced workplace violence during the past year. Approximately 76.81% felt that their professional value was half reflected by their salary. Approximately 75.32% once participated in anti-epidemic frontline work. Approximately 25.53% of participants felt very comfortable with publicity for healthcare workers concerning their nobility and dedication during the pandemic. Mean score of job enthusiasm was 39.14 ± 9.90, mean score of professional self-identity was 64.07 ± 15.61, and score of psychological support was 1.07 ± 0.74. See Table 2 in detail.

**TABLE 2:** Results of psychological measures of the study sample (*N* = 2,139).

**GAD-7 total score**	1.57 ± 2.95
Total < 10	2086 (97.52%)
Total ≥ 10	53 (2.48%)
Minimal	1849 (86.44%)
Mild	237 (11.08%)
Moderate	34 (1.59%)
Severe	19 (0.89%)
**PHQ-9 total score**	2.19 ± 3.80
Total < 10	2041 (95.41%)
Total ≥ 10	98 (4.58%)
Minimal	1740 (81.34%)
Mild	301 (14.07%)
Moderate	62 (2.90%)
Moderately severe	25 (1.17%)
Severe	11 (0.51%)
**Experience of workplace violence**	
No	1607 (75.13%)
Yes	532 (24.87%)
**Salary satisfaction**	
Reflected by half, 50%	1643 (76.81%)
Completely reflected, 100%	471 (22.02%)
Beyond one’s expectation, over 100%	25 (1.17%)
**Anti-epidemic participation**	
No	528 (24.68%)
Yes	1611 (75.32%)
**Do you wish your children to practice medicine before this pandemic?**	
Very reluctant	561 (26.23%)
Somewhat reluctant	303 (14.17%)
Neutral	701 (32.77%)
Somewhat willing	228 (10.66%)
Very willing	346 (16.18%)
**Do you wish your children to practice medicine after this pandemic?**	
Very reluctant	545 (25.48%)
Somewhat reluctant	319 (14.91%)
Neutral	679 (31.74%)
Somewhat willing	248 (11.59%)
Very willing	348 (16.27%)
**Media Publicity**	
Very uncomfortable	49 (2.29%)
Somewhat uncomfortable	232 (10.85%)
Neutral	463 (21.65%)
Somewhat comfortable	849 (39.69%)
Very comfortable	546 (25.53%)
**Job enthusiasm**	39.14 ± 9.90
**Professional self-identity**	64.07 ± 15.61
**Psychological support**	1.07 ± 0.74

*Continuous variables were summarized as mean ± standard deviation (S.D.), and categorical variables as number (percentage).*

### Independent *t*-Test of Seven-Item Generalized Anxiety Disorder and Nine-Item Patient Health Questionnaire

Female had higher score than male on GAD-7 (*t* = −2.13, *p* = 0.03) and PHQ-9 (*t* = −2.21, *p* = 0.03). Participants with experience of workplace violence scored higher than those without such experience on GAD-7 (*t* = −14.39, *p* < 0.001) and PHQ-9 (*t* = −15.40, *p* < 0.001). Participants who had not worked on the COVID-19 front line got higher scores of both GAD-7 (*t* = 2.50, *p* = 0.01) and PHQ-9 (*t* = 2.39, *p* = 0.02) than those had. See Table 3 for details.

**TABLE 3:** Independent *t*-test of seven-item generalized anxiety disorder (GAD-7) and nine-item patient health questionnaire (PHQ-9) by binary grouping variables.

	M ± S.D.	
Subgroups	GAD-7	PHQ-9
**Gender**		
Male	1.36 ± 2.79	1.91 ± 3.51
Female	1.65 ± 3.00*	2.30 ± 3.90*
**Experience of workplace violence**		
No	0.92 ± 2.11	1.31 ± 2.75
Yes	3.55 ± 4.04***	4.86 ± 5.08***
**Anti-epidemic participation**		
No	1.87 ± 3.26	2.55 ± 4.06
Yes	1.47 ± 2.84*	2.08 ± 3.70*

M, mean value; S.D., standard deviation; *p < 0.05, ***p < 0.001 vs. healthy volunteers; CI, confidence interval.

### One-Way ANOVA of Seven-Item Generalized Anxiety Disorder and Nine-Item Patient Health Questionnaire

One way ANOVA showed significant age effect of GAD-7 [*F*_(3,2138)_ = 4.99, *MSE* = 43.19, *p* < 0.01] and PHQ-9 [*F*_(3,2138)_ = 6.22, *MSE* = 89.09, *p* < 0.001]. *Post-hoc* test showed that participants aging from 31 to 40 scored higher than those aging from 20 to 30 on GAD-7 [*p* = 0.001, 95% *CI* (−0.97, −0.18)] and PHQ-9 [*p* < 0.001, 95% *CI* (−1.3, −0.27)] (Table 4).

**TABLE 4:** One-way ANOVA of seven-item generalized anxiety disorder (GAD-7) and nine-item patient health questionnaire (PHQ-9) by polytomous grouping variables.

	M ± S.D.	
Subgroups	GAD-7	PHQ-9
**Age**		
20–30	1.29 ± 2.60b	1.82 ± 3.41b
31–40	1.86 ± 3.15a	2.60 ± 4.16a
41–50	1.66 ± 3.11	2.36 ± 3.94
51–60	1.52 ± 3.21	1.83 ± 3.41
**Marital status**		
Unmarried	1.29 ± 2.54	1.96 ± 3.44
Married	1.66 ± 3.06	2.26 ± 3.92
Divorced	1.81 ± 3.40	2.41 ± 3.76
Widowed	3.33 ± 3.08	4.00 ± 3.74
**Educational level**		
Junior college	1.36 ± 3.00	1.78 ± 3.62b
Bachelor	1.65 ± 2.93	2.38 ± 3.91a,e
Master	2.31 ± 3.57e	2.59 ± 3.81
Doctor	0 ± 0	0 ± 0
Other	0.84 ± 2.02c	1.17 ± 2.38b
**Annual after-tax income**		
≤60,000	1.09 ± 2.39b,c	1.64 ± 3.22b
60,000–120,000	1.65 ± 3.04a	2.29 ± 3.91a
120,000–200,000	1.78 ± 3.08a	2.36 ± 3.88
≥200,000	0.38 ± 0.89	1.00 ± 2.22
**Department category**		
Internal medicine	1.86 ± 2.92e	2.61 ± 4.04e
Surgery	1.89 ± 3.32e	2.56 ± 4.17e
Gynecology and Pediatrics	1.84 ± 3.02	2.39 ± 3.88
Psychiatry	1.77 ± 4.25	2.26 ± 5.30
Others	1.22 ± 2.55a,b	1.78 ± 3.28a,b
**Title level**		
Primary title	1.21 ± 2.57b,c	1.69 ± 3.26b,c
Middle title	2.13 ± 3.50a	2.96 ± 4.52a
Vice senior title	1.84 ± 2.88a	2.55 ± 3.79a
Senior title	1.74 ± 2.79	2.54 ± 3.69

*M, mean value; S.D., standard deviation; In each group comparison, a, b, c, d, and e (if any) respectively denote significant difference from the first, second, third, fourth, or fifth group.*

Education effect of GAD-7 [*F*_(4,2138)_ = 3.75, *MSE* = 32.47, *p* < 0.01] or PHQ-9 [*F*_(4,2138)_ = 4.38, *MSE* = 62.73, *p* < 0.01] was significant. *Post-hoc* test showed that participants with master degree scored higher than those with other degree on GAD-7 [*p* = 0.01, 95% *CI* (0.22, 2.72)]. Participants with bachelor degree scored higher than those with junior college degree [*p* = 0.02, 95% *CI* (−1.14, −0.05)] or with other degree [*p* = 0.04, 95% *CI* (0.04, 2.38)] on PHQ-9 (Table 4).

Income effect of GAD-7 [*F*_(3,2138)_ = 4.82, MSE = 41.71, *p* < 0.01] or PHQ-9 [*F*_(3,2138)_ = 3.49, *MSE* = 50.12, *p* = 0.02] was significant. *Post-hoc* test showed that participants with income between 60,000 and 120,000 scored higher than those with income less than 60,000 on GAD-7 [*p* = 0.01, 95% *CI* (−1.03, −0.08)] and PHQ-9 [*p* = 0.03, 95% *CI* (−1.26, −0.04)]. Besides, participants with income between 120,000 and 200,000 scored higher than those with income less than 60,000 on GAD-7 [*p* = 0.01, 95% *CI* (−1.27, −0.11)] (Table 4).

Department category effect of GAD-7 [*F*_(4,2138)_ = 6.44, *MSE* = 55.49, *p* < 0.001] or PHQ-9 [*F*_(4,2138)_ = 5.39, *MSE* = 77.12, *p* < 0.001] was significant. *Post-hoc* test showed that participants from department of internal medicine scored higher than those from other department on GAD-7 [*p* < 0.001, 95% *CI* (0.24, 1.09)] and PHQ-9 [*p* = 0.001, 95% *CI* (0.22, 1.32)]. Participants from department of surgery scored higher than those from other department on GAD-7 [*p* < 0.01, 95% *CI* (0.11, 1.16)] and PHQ-9 [*p* < 0.01, 95% *CI* (0.15, 1.51)] (Table 4).

Title level effect of GAD-7 [*F*_(3,2138)_ = 14.94, MSE = 127.58, *p* < 0.001] or PHQ-9 [*F*_(3,2138)_ = 17.14, *MSE* = 241.66, *p* < 0.001] was significant. *Post-hoc* test showed that participants with middle title scored higher than those with primary title on GAD-7 [*p* < 0.001, 95% *CI* (−1.3, −0.55)] and PHQ-9 [*p* < 0.001, 95% *CI* (−1.76, −0.79)]. Participants with vice senior title scored higher than those with primary title on GAD-7 [*p* = 0.02, 95% *CI* (−1.20, −0.07)] and PHQ-9 [*p* = 0.01, 95% *CI* (−1.58, −0.14)] (Table 4).

However, marital status effect of GAD-7 or PHQ-9 score was insignificant according to one-way ANOVA (all *p*s > 0.05) (Table 4).

### Multiple Linear Regression Analyses of Seven-Item Generalized Anxiety Disorder and Nine-Item Patient Health Questionnaire

In regression, GAD-7 and PHQ-9 total scores served as dependent variables, respectively, and independent variables were age, gender, marital status, educational level, annual after-tax income, department category, job title, working age, experience of violence, salary satisfaction, anti-epidemic participation, media publicity, job enthusiasm, professional self-identity, and psychological support. See Table 5 in detail.

**TABLE 5:** Multiple linear regression (stepwise method) of seven-item generalized anxiety disorder (GAD-7) and nine-item patient health questionnaire (PHQ-9) by sociodemographic and supplementary variables.

	Healthcare workers (*n* = 2,139)				
	Associated factors	B	SE	β	*P*-value
GAD-7	Gender (Female)	0.37	0.13	0.06	<0.01
	Educational level (doctor)	−5.86	1.89	−0.06	<0.01
	Department category (Others)	−0.35	0.12	−0.06	<0.001
	Job title	0.22	0.07	0.06	<0.01
	Experience of workplace violence	2.23	0.14	0.33	<0.001
	Job enthusiasm	−0.03	0.01	−0.10	<0.001
	Professional self−identity	−0.02	0.01	−0.12	<0.001
PHQ−9	Gender (Female)	0.49	0.17	0.06	<0.01
	Educational level (doctor)	−8.07	2.39	−0.07	<0.01
	Department category (Others)	−0.37	0.15	−0.05	<0.05
	Job title	0.33	0.09	0.07	<0.001
	Experience of workplace violence	2.91	0.18	0.33	<0.001
	Job enthusiasm	−0.03	0.01	−0.07	<0.01
	Professional self−identity	−0.04	0.01	−0.16	<0.001
	Psychological support	−0.31	0.10	−0.06	<0.01

*β, standardized coefficients beta; B, unstandardized B; SE, coefficients standard error.*

According to the regression model of GAD-7 [*F*_(7,2138)_ = 75.76, *MSE* = 529.78, *p* < 0.001, adjusted *R* square = 0.20], the results showed that experience of violence (*t* = 16.13, *p* < 0.001), job title (*t* = 2.95, *p* < 0.01), and gender (*t* = 2.83, *p* < 0.01) was positively associated with GAD-7 score, while professional self-identity (*t* = −4.61, *p* < 0.001), job enthusiasm (*t* = −4.02, *p* < 0.001), department category (others *vs*. psychiatry) (*t* = −3.04, *p* < 0.01), and educational level (junior college *vs*. doctor) (*t* = −3.10, *p* < 0.01) were negatively associated with GAD-7 score. However, age (*t* = 0.94, *p* = 0.35), marital status (all *p*s > 0.05), annual after-tax income (*t* = 1.20, *p* = 0.23), working age (*t* = 1.15, *p* = 0.25), salary satisfaction (*t* = −1.10, *p* = 0.27), anti-epidemic participation (*t* = −0.99, *p* = 0.32), media publicity (*t* = −1.79, *p* = 0.07), and psychological support (*t* = −1.15, *p* = 0.25) were not associated with GAD-7 score.

Besides, according to the regression model of PHQ-9 [*F*_(8,2138)_ = 79.05, *MSE* = 882.16, *p* < 0.001, adjusted *R* square = 0.23], the results showed that gender (*t* = 2.98, *p* < 0.01), job title (*t* = 3.54, *p* < 0.001), and experience of violence (*t* = 16.57, *p* < 0.001) were positively associated with PHQ-9 score, while professional self-identity (*t* = −6.40, *p* < 0.001), psychological support (*t* = −3.01, *p* = 0.003), educational level (junior college *vs*. doctor) (*t* = −3.38, *p* = 0.001), job enthusiasm (*t* = −2.88, *p* < 0.01), and department category (others *vs*. psychiatry) (*t* = −2.52, *p* = 0.01) were negatively associated with the PHQ-9 scores. However, age (*t* < 0.01, *p* = 1.00), marital status (all *p*s > 0.10), annual after-tax income (*t* = 0.67, *p* = 0.51), working age (*t* = 0.61, *p* = 0.54), job enthusiasm (*t* = 0.50, *p* = 0.62), anti-epidemic participation (*t* = −0.64, *p* = 0.52), and media publicity (*t* = −1.89, *p* = 0.06) were not associated with the PHQ-9 scores.

## Discussion

In the current study, we found that participants with female gender, with experience of workplace violence, without participation in anti-epidemic front-line work during pandemic, aging from 31 to 40, with higher educational level, with middle level of annual after-tax income, from department of internal medicine or surgery, or with middle level of job title scored higher on both GAD-7 and PHQ-9, which was partly in line with our first hypothesis. Regression analysis showed that female gender, high job title, and experience of workplace violence were positively associated with anxiety or depression. Doctoral education, other department, job enthusiasm, and professional self-identity were negatively associated with anxiety or depression. Additionally, psychological support was negatively associated with depression. Thus, our second hypothesis was confirmed.

Based on univariate analysis, participants aged 31–40 scored higher on GAD-7 and PHQ-9. We speculated that healthcare workers of such age usually had young children and living parents in their families, thus they had the greatest concern regarding viral transmission to their families (Cai et al., 2020). Participants with middle-income level scored higher on anxiety or depression, probably because people of medium income level were in a period of rising careers, hence subjectively feeling more pressured and more prone to emotional problems. Previous research mainly focused on the connection between income reduction and emotional state (Xing et al., 2020; Peng et al., 2021). Therefore, the impact of income level needs to be further verified by follow-up research. Participants who had not joined in anti-epidemic front-line work scored higher on GAD-7 and PHQ-9. We speculated that participants once joining in front-line work were experienced in and accustomed to the virus. In turn, participants without such experience might go to the front-line in the future, and they might feel fear, anxious, and depressed about the unknown. However, age, income, and anti-epidemic participation were not significant in the regression analysis, so these factors might be less important.

Combining univariate and multivariate analysis, female gender and middle-job title were positively associated with both anxiety and depression, which was consistent with previous result (Lai et al., 2020). As to education, group analysis showed that participants with bachelor or master degrees had higher anxiety or depression, while regression analysis showed that doctoral education was negatively associated with anxiety or depression. Considering a number of people with doctorate degrees in our sample were negligible, this regression result might not be stable. Therefore, our study generally supported high educational level contributed to emotional problems, which accorded with previous findings (Mo et al., 2020; Wang et al., 2021). Participants from the department of internal medicine or surgery scored higher on anxiety and depression, which was consistent with the regression result that other department was a negative associated factor of emotional issues. These results were similar to a previous study in the United States (Sonis et al., 2021), and that surgical department had significantly higher rates of self-reported depression and anxiety (Louie et al., 2020; Jemal et al., 2021).

In terms of psychosocial factors, the experience of workplace violence was positively associated with both anxiety and depression, which was explained by that healthcare workers who experienced physical and non-physical violence were more likely to suffer from depression and anxiety symptoms than those not (Shi et al., 2020).

We also found that both job enthusiasm and professional self-identity were negatively associated with anxiety and depression, which is poorly studied at present. Instead, previous research generally demonstrated emotional problems could lead to poor vocational dedication and job enthusiasm. For example, concurrent depression and anxiety, along with uncertainty and burden in the workplace, pre-disposed individuals to professional burnout, and perception of reduced accomplishment (Albott et al., 2020). And professional burnout was accompanied by negative attitudes and disengagement from work (Demerouti et al., 2003; Malach-Pines, 2005), sign of reduced job enthusiasm. Therefore, our study showed that vocational evaluation could in turn influence the emotional problems.

Moreover, psychological support was negatively associated with depression, which means depressive symptoms of healthcare workers could be relieved by mental health service offered by the work unit. Previous literature has called for early assessment of healthcare workers’ mental health and appropriate psychological interventions (Li et al., 2020; Xiang et al., 2020), and our result supported the necessity of this proposal.

Another two psychosocial factors, salary satisfaction and media publicity, were insignificant in regression analysis, which means healthcare workers’ expectation of reward and social media exposure might make no difference to psychological health, at least during this pandemic. Comparatively, the experience of medical violence, job enthusiasm, professional self-identity, and psychological support are far more critical to healthcare workers’ mental health.

However, this study has several limitations. First, all participants were recruited from Jiande City, thus limiting the generalization of our findings to other regions. Second, this was a cross-sectional self-report study. With the normalization of anti-epidemic work, the mental health symptoms of healthcare workers could vary. Therefore, a longitudinal follow-up study could have offered a temporal change of the emotional state of healthcare workers. Third, this study did not distinguish preexisting emotional symptoms from new symptoms resulting from the COVID-19 pandemic, and this might be a confounding factor. Fourth, PHQ-9 and GAD-7 are simple screening tools and the standard diagnostic tools would have offered more accurate results. Last, an alpha error might be inflated in our study since many statistical analyses were conducted.

Nevertheless, we have found the emotional state of healthcare workers is influenced by multiple sociodemographic variables and vocational evaluation. Our study revealed that female gender, high educational level, medium job title, and experience of workplace violence were risk factors of anxiety or depression. Non-mainstream department, job enthusiasm, professional self-identity, and psychological support were protective factors of anxiety or depression. Self-tailored psychological intervention should be based on the predisposing factors above to mentally prepare healthcare workers for this long-lasting battle against COVID-19.

## Data Availability Statement

The raw data supporting the conclusions of this article will be made available by the authors, without undue reservation.

## Ethics Statement

The studies involving human participants were reviewed and approved by Ethics Committee of the Fourth People’s Hospital of Jiande City. The patients/participants provided their written informed consent to participate in this study.

## Author Contributions

MJ, XS, WC, and JT conceived the study. MJ, XS, SR, YL, ZP, YS, SZ, LY, and HW contributed to the study design and collected the data. MJ, XS, and YL analyzed the data. MJ, XS, YL, WC, and JT drafted the manuscript. All authors have read and approved the final manuscript.

## Conflict of Interest

The authors declare that the research was conducted in the absence of any commercial or financial relationships that could be construed as a potential conflict of interest.

## Publisher’s Note

All claims expressed in this article are solely those of the authors and do not necessarily represent those of their affiliated organizations, or those of the publisher, the editors and the reviewers. Any product that may be evaluated in this article, or claim that may be made by its manufacturer, is not guaranteed or endorsed by the publisher.
