# Supplementary material for: Emotional State of Chinese Healthcare Workers During COVID-19 Pandemic
Source: Front Psychol. 2022 Mar 23;13:854815. doi: 10.3389/fpsyg.2022.854815 (PMC8984149; doi:10.3389/fpsyg.2022.854815)
Supplement: Supplementary file 1 [file Table_1.docx]

**Psychosocial questions related to vocation and the pandemic**

| **A. Experience of workplace violence**  Options:  1—Yes; 2—No  Question:  Have you experienced following violent acts against healthcare workers during the past 12 months? |
| --- |
| 1. Verbal violence (sarcasm, roar, or abuse) |
| 2. Patients making difficulties (nitpicking, unreasonable request, or poor adherence) |
| 3. Reputation damage (unreasonable complaint) |
| 4. Destructive behaviors (destroying public facilities, gathering a crowd and making disturbances, or malicious photographing) |
| 5. Being threatened (verbal or written threat, with weapon in hand, fist shaking, or stalking) |
| 6. Direct physical harm (being bitten, pushed over, beaten up, chopped, or smashed) |
| 7. Sexual assault (physical contact in sexual body parts) |
| **B.** **Salary satisfaction** |
| Options:  1—Reflected by half, 50%; 2—Completely reflected, 100%; 3—Beyond one’s expectation, over 100%  Question:  To what extent do you think your professional value is reflected by your salary? |
| **C. Anti-epidemic participation**  Options:  1—Yes; 2—No  Question:  Have you participated in front-line work against COVID-19? |
| **D. Media publicity**  Options:  1—Very uncomfortable; 2—Somewhat uncomfortable; 3—Neutral; 4—Somewhat comfortable; 5—Very comfortable  Question:  How do you feel about the national, media, or medical publicity for healthcare workers’ noble sense of morality and mission (e.g., angels in white, anti-epidemic heroes)? |
| **E. Job enthusiasm**  Options:  1—Strongly disagree; 2—Somewhat disagree; 3—Neither agree or disagree; 4—Somewhat agree; 5—Strongly agree  Statements:  1. I like my work itself.  2. I find my work interesting.  3. I find my work appealing.  4. I enjoy my work.  5. If I participate in anti-epidemic work, I will earn satisfactory economic rewards, such as awards or bonus.  6. If I participate in anti-epidemic work, it will benefit my promotion or career development.  7. If I participate in anti-epidemic work, it will benefit my performance evaluation.  8. If I participate in anti-epidemic work, I will win my senior’s approval.  9. If I participate in anti-epidemic work, I will win my colleague’s approval.  10. If I participate in anti-epidemic work, I will get satisfactory welfare in return.  11. If I participate in anti-epidemic work, I will receive public praise from senior leaders or senior management. |
| **F. Professional self-identity**  Options:  1—Strongly disagree; 2—Disagree; 3—Somewhat disagree; 4—Neither agree or disagree; 5—Somewhat agree; 6—Agree; 7—Strongly agree  Statements:  1. Generally speaking, when someone praises healthcare workers, it feels like a personal compliment to me.  2. Generally speaking, when someone criticizes healthcare workers, it feels like a personal insult to me.  3. When I talk about healthcare workers, I usually say “we” instead of “they”.  4. Medical success is my personal success.  5. When media report criticizes healthcare workers, I feel awkward.  6. Generally speaking, my organization is fair to me.  7. Generally speaking, I can count on this organization.  8. Generally speaking, I am treated fairly here.  9. My life is close to my ideal in most aspects.  10. I have a good life condition.  11. I am satisfied with my life.  12. I have already got what I want in my life so far.  13. If I could live once again, basically I wound not make substantial changes. |
| **G. Psychological support**  Options (multiple choice):  1—lecture on mental health; 2—mental counselling; 3—other kind  Question:  What free or low-cost psychological services has the work unit offered to healthcare workers? |
